# 6-Methoxyluteolin from the Invasive Plant *Tithonia diversifolia* Suppresses Melanization and Impairs Epidermal Integrity in *Spodoptera litura* Larvae

**DOI:** 10.3390/insects17060535

**Published:** 2026-05-22

**Authors:** Zhandi Wang, Junying Liu, Yandan Wang, Weili Dai, Yinglan Dai, Lin Jia, Weifeng Ding

**Affiliations:** 1 School of Agronomy and Biological Science, Yuxi Normal University, Yuxi 653100, China; zhdwang@yxnu.edu.cn (Z.W.); jyliu@yxnu.edu.cn (J.L.); 2023142242@yxnu.edu.cn (Y.W.); 2022142204@yxnu.edu.cn (W.D.); 2023144130@yxnu.edu.cn (Y.D.); jl@yxnu.edu.cn (L.J.); 2 School of Biological and Chemical Science, Pu’er University, Pu’er 665000, China

**Keywords:** *Tithonia diversifolia*, *Spodoptera litura*, 6-methoxyluteolin, melanin biosynthesis inhibition, tyrosinase, cuticle, plant-derived insecticide, lepidoptera pest management

## Abstract

The invasive plant *Tithonia diversifolia* is a promising source of botanical insecticides, yet the mechanisms by which its active compounds impair insect physiology remain incompletely characterized. This study investigated the effects of 6-methoxyluteolin (6-ML), a flavonoid isolated from *T. diversifolia*, on melanization and cuticle integrity in *Spodoptera litura* larvae. Cuticle melanization, mediated by tyrosinase-catalyzed oxidation of tyrosine-derived substrates, is essential for cuticle sclerotization and immune defense in insects. Whether 6-ML directly targets this pathway has not been previously examined at the biochemical and histological level. Tyrosinase activity and melanin content were measured across 3rd–6th-instar larvae exposed to a concentration gradient of 6-ML (1.625–100 μg/mL). Histological sections of 4th- and 5th-instar larvae were prepared by paraffin embedding and hematoxylin and eosin (H&E) staining. 6-ML significantly reduced tyrosinase activity and melanin content in a concentration-dependent manner across all instars examined. Histological analysis revealed progressive cuticle thinning and loss of pigmentation granules with increasing 6-ML concentration. These findings provide the first biochemical and histological evidence that 6-ML targets the melanization pathway in *S. litura*, supporting its potential as a melanization-inhibiting botanical insecticide.

## Simple Summary

Invasive plants often produce chemical compounds that help them compete in new environments, and some of these compounds can harm insect pests. Understanding how these compounds affect insects could help us develop safer, more environmentally friendly alternatives to conventional chemical pesticides. In this study, we investigated how a compound called 6-methoxyluteolin, extracted from the invasive plant *Tithonia diversifolia* (Mexican sunflower), affects the integument of the agricultural pest *Spodoptera litura* (common cutworm) at different stages of larval development. We found that this compound reduced the activity of a key pigment-forming enzyme and decreased melanin levels in the larval integument across multiple developmental stages. Histological analysis further revealed progressive thinning and structural disorganization of the outer cuticle layer with increasing compound concentration. These results suggest that 6-methoxyluteolin disrupts the melanization process essential for cuticle integrity in insect larvae, and support its potential as a targeted botanical insecticide.

## 1. Introduction

The common cutworm *Spodoptera litura* (Fabricius) (Lepidoptera: Noctuidae) is one of the most economically destructive polyphagous pests worldwide, capable of infesting over 100 host plant species across diverse agricultural ecosystems [1]. Reliance on synthetic chemical insecticides for its management has led to widespread resistance and concerns over environmental contamination, driving demand for alternative, ecologically sustainable control strategies [2,3]. Plant-derived bioactive compounds represent a promising reservoir of novel insecticides, owing to their chemical diversity, biodegradability, and multifaceted modes of action [3].

*Tithonia diversifolia* (Hemsl.) A. Gray (Asteraceae), an invasive weed widely distributed across tropical and subtropical regions, including Yunnan Province, China, has attracted growing attention as a source of insecticidal secondary metabolites [4]. Previous investigations from our group characterized the phytochemical composition of *T. diversifolia* methanol extracts and demonstrated significant insecticidal and growth-inhibitory activity against *S. litura* larvae, with 6-methoxyluteolin (6-ML), hispidulin, and 1,5-quinic acid identified as principal active compounds [5]. Transcriptomic profiling of extract-treated larvae further indicated enrichment of differentially expressed genes associated with cuticle structural constituents, consistent with a mode of action targeting epidermal physiology [5]. However, as that study employed a complex multi-component extract, the independent contribution of 6-ML to epidermal disruption could not be resolved, and no biochemical or histological evidence of cuticle impairment by the isolated compound was provided.

The insect cuticle is a dynamic, multi-layered extracellular matrix essential for mechanical protection, water retention, immune defense, and developmental integrity [6,7]. Melanization of the cuticle, mediated by the tyrosine metabolic cascade, is a conserved process contributing to cuticle sclerotization, wound repair, and innate immune responses [8,9]. The key enzyme tyrosinase (phenoloxidase) catalyzes the hydroxylation of tyrosine to dihydroxyphenylalanine (DOPA) and its subsequent oxidation to dopaquinone, initiating the biosynthetic pathway leading to melanin deposition [8,10]. Disruption of this pathway impairs cuticle integrity and immune competence: silencing of tyrosine hydroxylase in *Henosepilachna vigintioctopunctata* resulted in cuticle tanning failure and 100% larval mortality [11], while knockdown of melanin synthesis genes in *Blattella germanica* produced pale, softened cuticles with elevated permeability [12].

Flavonoids are well-established inhibitors of tyrosinase activity across diverse biological contexts [13,14], and the structural features of luteolin-type flavonoids, including the catechol B-ring and 4′-hydroxyl group, are associated with copper chelation at the enzyme active site [14]. In particular, methoxy substituents on the flavone scaffold have been shown to direct binding toward the hydrophobic substrate-binding pocket of tyrosinase, potentially enhancing inhibitory potency [14]. A related botanical compound, camptothecin, was recently shown to inhibit laccase activity and impair cuticle tanning in *Ostrinia furnacalis*, providing precedent for plant-derived compounds targeting the melanization machinery [15]. However, the capacity of 6-ML specifically to suppress insect melanization in vivo across multiple developmental instars, and the consequent histological effects on cuticle architecture, have not been investigated.

In the present study, we provide biochemical and histological evidence that 6-ML derived from *T. diversifolia* suppresses tyrosinase activity and melanin biosynthesis in *S. litura* larvae across the 3rd to 6th instars, leading to progressive cuticle thinning and structural disorganization. These results establish melanization pathway disruption as a primary mode of action of 6-ML and support its development as a lead botanical insecticide.

## 2. Materials and Methods

### 2.1. Insect Rearing and Treatment

*Spodoptera litura* larvae were reared under controlled laboratory conditions (25 ± 1 °C, 70 ± 5% relative humidity, 16:8 h light:dark photoperiod) on artificial diet. 6-Methoxyluteolin (6-ML; purity ≥ 98% by HPLC, Macklin Biochemical Co., Ltd., Shanghai, China; lot no. C18244850) was dissolved in methanol and applied to the surface of the artificial diet following the formulation described by Wang et al. [5] at concentrations of 1.625, 3.125, 6.25, 12.5, 25, 50, and 100 μg/mL, following air-drying to remove solvent. Control larvae received equivalent solvent treatment. Second-instar larvae (7 days post-hatching) were allocated to treatment groups (30 larvae per group) and maintained in individual Petri dishes. Larvae were fed the treated artificial diet for three days, after which the diet was replaced with untreated standard diet (without compound or solvent) for the remainder of the rearing period. Three independent biological replicates were conducted for each treatment group.

### 2.2. Tyrosinase Activity Assay

Larvae at the 3rd, 4th, 5th, and 6th instars (*n* = 3 per group) were homogenized in 3 volumes of 0.1 M phosphate buffer (pH 7.0; Shengshi (Xiamen) Standard Substance Technology Co., Ltd., Xiamen, China; lot no. N20251013) on ice and centrifuged at 12,000 rpm for 30 min at 4 °C using a GTR420C high-speed centrifuge (Kecheng Instrument Co., Ltd., Changsha, China). The supernatant (10 μL) was mixed with 140 μL phosphate buffer and 50 μL catechol substrate (Solarbio Science & Technology Co., Ltd., Beijing, China; lot no. A2508116), and absorbance at 475 nm was recorded every 40 s for 4 min using a SpectraMax Plus 384 microplate reader (Molecular Devices LLC, San Jose, CA, USA). Enzyme activity was expressed as U/mg protein, with total protein concentration determined by the Bradford method using Coomassie Brilliant Blue G-250 (Beijing Bioleader Technology Co., Ltd., Beijing, China; CAS no. 6104-58-1).

### 2.3. Melanin Content Determination

Following treatment, larvae at the 3rd, 4th, 5th, and 6th instars were randomly selected (*n* = 5 per group), and the integument was carefully dissected on ice, weighed, and homogenized in 9 volumes of 1 M NaOH (Tianjin Fengchuan Chemical Reagent Science Co., Ltd., Tianjin, China). Homogenates were boiled for 20 min, centrifuged at 10,000 rpm for 5 min using a GTR420C high-speed centrifuge (Kecheng Instrument Co., Ltd., Changsha, China), and the absorbance of the supernatant was measured at 405 nm. Melanin content was quantified against a standard curve prepared from serial dilutions of synthetic melanin standard (200–800 μg/mL in 1 M NaOH; Macklin Biochemical Co., Ltd., Shanghai, China; lot no. M10027442) and expressed as μg/mg tissue.

### 2.4. Histological Analysis of Larval Cuticle

Larvae from the 4th and 5th instars were fixed in 4% paraformaldehyde (Aladdin Biochemical Technology Co., Ltd., Shanghai, China; lot no. 00133991) at 4 °C for 12 h, washed under running water for 4 h, dehydrated through a graded ethanol series (30–100%; Chengdu Kelong Chemical Co., Ltd., Chengdu, China; lot no. 2023101001), cleared in xylene (Tianjin Zhiyuan Chemical Reagent Co., Ltd., Tianjin, China; lot no. 20241201), and embedded in paraffin (Sinopharm Chemical Reagent Co., Ltd., Shanghai, China; lot no. 20220811). Serial sections of 6–8 μm were cut and mounted on albumin-glycerol-coated slides (3:1 *v*/*v*). Sections were deparaffinized, rehydrated, and stained with hematoxylin (Solarbio Science & Technology Co., Ltd., Beijing, China; lot no. A729R031) and eosin (Tianjin Guangfu Fine Chemical Research Institute, Tianjin, China) (H&E) following standard protocols. Cuticle morphology was assessed from bright-field micrographs, with observations made across three individuals per treatment group.

### 2.5. Statistical Analysis

All data are expressed as mean ± standard error of the mean (SEM) from three independent biological replicates. Statistical significance among treatment groups was assessed by one-way ANOVA followed by Tukey’s multiple comparison test using SAS 9.4 software (SAS Institute Inc., Cary, NC, USA). Prior to ANOVA, data normality was assessed using the Shapiro–Wilk test and homogeneity of variances was confirmed using Levene’s test; all datasets met the assumptions required for parametric analysis (*p* > 0.05 for both tests). Differences were considered statistically significant at *p* < 0.05, and significance groupings are indicated by lowercase letters in all figures.

## 3. Results

### 3.1. 6-ML Perturbs Tyrosinase Activity Across Multiple Larval Instars

**Figure 1:**
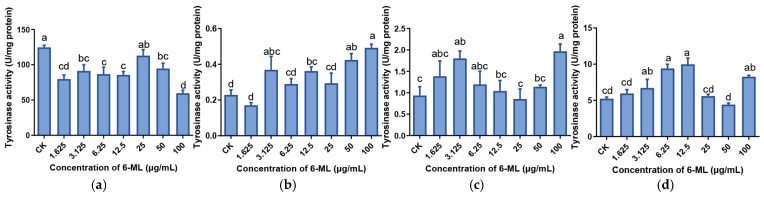

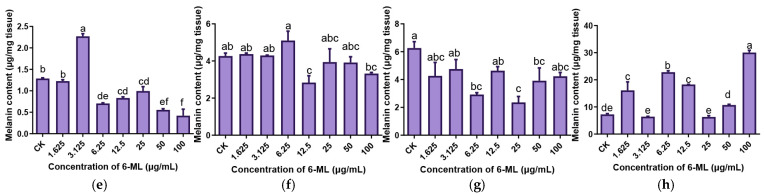
Effects of 6-methoxyluteolin (6-ML) on tyrosinase activity and melanin content in *Spodoptera litura* larvae at different developmental instars. (**a**–**d**) Tyrosinase activity in 3rd-, 4th-, 5th-, and 6th-instar larvae, respectively. (**e**–**h**) Melanin content in 3rd-, 4th-, 5th-, and 6th-instar larvae, respectively. Data are presented as mean ± SEM (*n* = 3 biological replicates). Different lowercase letters above bars indicate statistically significant differences among treatment groups (one-way ANOVA followed by Tukey’s post hoc test, *p* < 0.05). CK, solvent control.

Tyrosinase activity was measured across 3rd- to 6th-instar larvae following 6-ML exposure (Figure 1a–d). In 3rd-instar larvae, the control group showed the highest activity (approximately 125 U/mg protein), and treatment significantly reduced activity at the majority of concentrations tested, with the lowest value recorded at 100 μg/mL (*p* < 0.05; Figure 1a). In 4th-instar larvae, baseline activity in the control group was markedly lower than in 3rd instar, and significant suppression was observed at 1.625 μg/mL, with partial variation at higher doses (Figure 1b). In 5th- and 6th-instar larvae, tyrosinase activity showed concentration-dependent variation with significant differences detected among treatment groups at both instars (Figure 1c,d). Across all instars, 6-ML treatment consistently perturbed tyrosinase activity relative to controls, indicating disruption of the melanization enzyme system throughout larval development.

### 3.2. 6-ML Reduces Melanin Content in the Larval Integument

**Figure 2:**
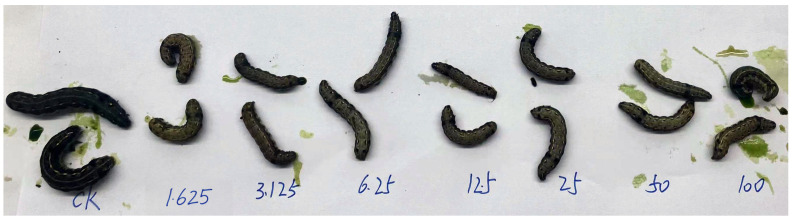
Macroscopic appearance of *Spodoptera litura* larvae following 6-methoxyluteolin (6-ML) treatment at increasing concentrations. Representative larvae from each treatment group (CK, 1.625, 3.125, 6.25, 12.5, 25, 50, and 100 μg/mL) were photographed simultaneously under identical lighting conditions to minimize color deviation across groups. Labels indicate the corresponding 6-ML concentration (μg/mL); CK denotes the solvent control. Progressive lightening of larval body coloration is evident with increasing 6-ML concentration.

Melanin content in the larval integument showed concentration-associated variation following 6-ML treatment across all four instars examined, with suppressive effects most pronounced in 3rd-instar larvae (Figure 1e–h). In 3rd-instar larvae, melanin content declined progressively with increasing concentration, from a maximum of approximately 2.2 μg/mg tissue at 3.125 μg/mL to a minimum of approximately 0.4 μg/mg tissue at 100 μg/mL, representing a greater than 5-fold reduction (*p* < 0.05; Figure 1e). In 4th-instar larvae, significant suppression was most evident at 12.5 μg/mL relative to the control (Figure 1f). In 5th-instar larvae, the control group showed the highest melanin content (approximately 6.2 μg/mg tissue), and significant reductions were detected at 6.25 and 25 μg/mL (Figure 1g). In 6th-instar larvae, melanin content also varied significantly across concentrations, with the lowest values observed at 25 and 3.125 μg/mL (Figure 1h). Overall, these data demonstrate that 6-ML consistently suppresses melanin accumulation in *S. litura* larvae across multiple developmental stages, with 3rd-instar larvae showing the most pronounced response. Macroscopic observation of treated larvae further corroborated these biochemical findings. As shown in Figure 2, control larvae (CK) displayed characteristic dark pigmentation with well-defined dorsal stripe patterning, whereas larvae exposed to increasing concentrations of 6-ML exhibited progressive lightening of body coloration. Visible depigmentation was apparent from 3.125 μg/mL onward, with larvae at 25–50 μg/mL showing markedly pale, yellow-green integuments compared to controls. These external phenotypic changes are consistent with the suppression of melanin accumulation quantified biochemically, and provide direct visual evidence of 6-ML-induced disruption of integumental pigmentation in *S. litura* larvae.

### 3.3. Histological and Morphometric Analysis Reveals Concentration-Dependent Cuticle Thinning and Depigmentation

**Figure 3:**
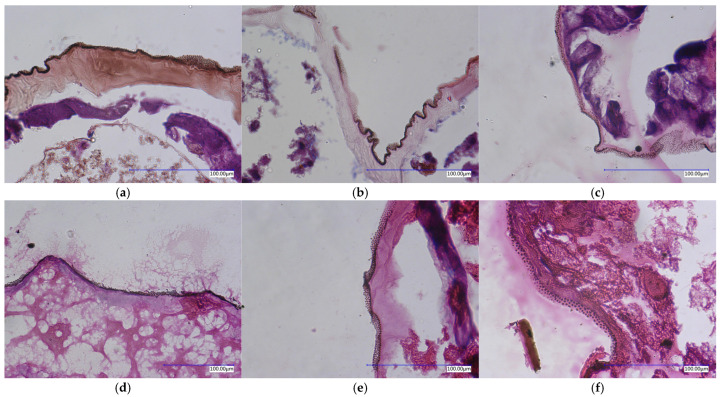
Histological sections of the dorsal cuticle of 4th and 5th-instar *Spodoptera litura* larvae following 6-methoxyluteolin (6-ML) treatment, stained with hematoxylin and eosin (H&E). (**a**–**c**) 4th-instar larvae: (**a**) solvent control (CK), showing dense melanin granule deposition along the outer cuticle surface; (**b**) 12.5 μg/mL, showing visible loosening of cuticle structure with partial reduction in melanin granule density; (**c**) 100 μg/mL, showing dispersed melanin granule distribution and structural disorganization. (**d**–**f**) 5th-instar larvae: (**d**) solvent control (CK), showing dense and continuous melanin granule arrangement; (**e**) 6.25 μg/mL, showing loosened cuticle structure with reduced melanin granule deposition and lightened pigmentation; (**f**) 25 μg/mL, showing marked structural loosening with notably reduced melanin granule accumulation. Progressive loss of pigmentation granules and structural disorganization of the cuticle layer are evident with increasing 6-ML concentration. Scale bars: 100 μm for all panels.

H&E-stained paraffin sections of 4th- and 5th-instar larvae treated with 6-ML revealed concentration-dependent alterations in cuticle architecture (Figure 3). In 4th instar control larvae, the dorsal cuticle appeared as a continuous layer with dense melanin granule deposition along the outer surface (Figure 3a). At 12.5 μg/mL, the cuticle structure showed visible loosening with reduced pigmentation granule density (Figure 3b). At 100 μg/mL, the cuticle layer was markedly attenuated, with extensive loss of pigmentation granules and structural disorganization (Figure 3c), providing direct morphometric evidence of cuticle thinning. In 5th-instar control larvae, the cuticle similarly presented as a well-defined, pigmented layer (Figure 3d). At 6.25 μg/mL, the cuticle showed visible loosening with reduced melanin granule deposition and lightened pigmentation (Figure 3e). At 25 μg/mL, structural fragmentation and irregular granule distribution were apparent (Figure 3f). Collectively, these histological observations demonstrate that 6-ML induces concentration-dependent loss of melanin granules and impairs cuticle structural integrity across both larval instars examined, consistent with disruption of the melanization-dependent cuticle sclerotization process.

To provide quantitative support for the observed morphological changes, cuticle thickness was measured from cross-sectional micrographs of 4th-instar larvae across all treatment concentrations (Table 1). Control larvae exhibited a mean cuticle thickness of 4.70 ± 0.13 μm, which was significantly greater than all 6-ML-treated groups (*p* < 0.05). The greatest reduction was observed at 12.5 μg/mL (2.53 ± 0.13 μm), representing a 46% decrease relative to controls. Concentrations of 6.25 μg/mL and 12.5 μg/mL produced the most pronounced thinning (group c), while higher concentrations (25–100 μg/mL) also resulted in significant reductions relative to controls (group b). These morphometric data confirm that 6-ML induces significant structural thinning of the larval cuticle in a statistically supported manner, independently of the concentration–response patterns observed in the enzymatic assays.

## 4. Discussion

This study provides biochemical and histological evidence that 6-methoxyluteolin (6-ML) from the invasive plant *Tithonia diversifolia* disrupts melanization and impairs epidermal integrity in *Spodoptera litura* larvae. The convergent findings across two complementary lines of evidence—perturbation of tyrosinase activity and melanin content, and progressive cuticle structural disorganization—are consistent with the interpretation that 6-ML targets the tyrosine-mediated melanization pathway as a primary mode of insecticidal action.

The melanization cascade is central to cuticle formation and immune defense in insects [8,9]. Tyrosinase catalyzes the rate-limiting steps of melanin biosynthesis, and its inhibition has been shown to produce severe developmental and physiological consequences across diverse insect species [11,12]. In the present study, 6-ML treatment significantly perturbed tyrosinase activity across all instars examined, with the most pronounced inhibitory effect observed in 3rd-instar larvae; later instars exhibited more complex concentration–response relationships, potentially reflecting compensatory regulatory mechanisms. The reduction in melanin content observed in multiple instars, most consistently in 3rd-instar larvae, is consistent with the interpretation that enzymatic perturbation of the melanization pathway translates into altered melanin deposition in the integument. These results are consistent with the well-documented tyrosinase-inhibitory activity of flavonoids bearing the catechol-type B-ring characteristic of luteolin derivatives [13,14].

The histological data provide direct tissue-level validation of the biochemical findings. Paraffin sections of 4th- and 5th-instar larvae revealed concentration-dependent loss of pigmentation granules and structural disorganization of the cuticle layer at doses as low as 6.25 μg/mL. Quantitative morphometric analysis further confirmed that cuticle thickness in 4th-instar larvae was significantly reduced across all treatment concentrations relative to controls (4.70 ± 0.13 μm in CK vs. 2.53 ± 0.13 μm at 12.5 μg/mL; Table 1), providing objective evidence of cuticle thinning that complements the histological observations. These features are consistent with impaired cuticle sclerotization, a process that depends on melanin precursor-mediated crosslinking of cuticular proteins [8]. Similar histological phenotypes have been described following RNAi-mediated silencing of tyrosine hydroxylase in *H. vigintioctopunctata* [11] and pharmacological inhibition of cuticle tanning in *O. furnacalis* [15], reinforcing the conclusion that melanization pathway disruption is the primary mechanism of cuticle impairment observed here.

A notable feature of the present findings is that 6-ML effects were detectable across four consecutive larval instars (3rd–6th), suggesting persistent inhibitory activity throughout larval development. It is notable that these effects were observed following a three-day exposure period initiated at the second instar, suggesting that even brief early-stage exposure to 6-ML is sufficient to induce persistent disruption of the melanization pathway across subsequent developmental stages. The relatively greater sensitivity of 3rd-instar larvae may reflect heightened cuticle synthesis activity and active melanization associated with early larval stages following each molt [8]. The complex concentration-response patterns observed in some instars may reflect compensatory regulatory responses to enzyme inhibition, as has been reported for other flavonoid-enzyme interactions [13,14]. Notably, while the enzymatic and melanin content data exhibited instar-dependent variation in response magnitude, the morphometric evidence from 4th-instar larvae provided unambiguous quantitative confirmation of cuticle thinning (Table 1), supporting the biological relevance of 6-ML-induced integumental disruption regardless of the non-monotonic enzymatic patterns observed in later instars.

These findings complement and extend previous work from our group, which identified 6-ML as an insecticidally active compound in *T. diversifolia* extracts and observed enrichment of cuticle-associated gene categories in transcriptomic profiles of extract-treated larvae [5]. The present study establishes that 6-ML alone is sufficient to reproduce the epidermal phenotype and provides the first biochemical and histological evidence linking 6-ML exposure directly to melanization pathway disruption. This mechanistic specificity distinguishes 6-ML from many botanical insecticides whose modes of action remain broadly defined.

From an applied perspective, the targeting of the melanization pathway represents an attractive strategy for botanical insecticide development, as melanization is insect-specific and absent in vertebrates, reducing the likelihood of non-target toxicity [9]. Future studies should conduct kinetic enzyme inhibition assays to characterize the mode of 6-ML inhibition, quantify effects on cuticle mechanical properties and permeability, and evaluate activity under field-relevant conditions.

## 5. Conclusions

This study demonstrates that 6-methoxyluteolin (6-ML) from *Tithonia diversifolia* suppresses tyrosinase activity and melanin content in *Spodoptera litura* larvae across the 3rd to 6th instars, and induces progressive histological deterioration of the larval cuticle, including depigmentation and structural disorganization. Collectively, these findings provide the first biochemical and histological evidence for melanization pathway disruption as a primary mode of action of 6-ML in lepidopteran larvae, and support its potential as a lead compound for the development of melanization-targeting botanical insecticides against *S. litura* and related pests.

## Figures and Tables

**Table 1:** Cuticle thickness of the dorsal integument in 4th instar *Spodoptera litura* larvae following 6-methoxyluteolin (6-ML) treatment at increasing concentrations.

Concentration (μg/mL)	*n*	Mean Thickness (μm)	SEM	Significance
CK	29	4.70	0.13	a
1.625	29	3.39	0.16	b
3.125	28	3.19	0.07	b
6.25	29	2.81	0.11	c
12.5	31	2.53	0.13	c
25	21	3.33	0.09	b
50	25	3.22	0.09	b
100	26	3.20	0.09	b

Data are presented as mean ± SEM. *n* = number of individual cross-sectional measurements from three biological replicates. Different letters in the Significance column indicate statistically significant differences among treatment groups (one-way ANOVA followed by Tukey’s post hoc test, *p* < 0.05). CK, solvent control.

## Data Availability

The datasets generated and analyzed during the current study are available from the corresponding author upon reasonable request.
